# High hepatitis B virus load is associated with hepatocellular carcinomas development in Chinese chronic hepatitis B patients: a case control study

**DOI:** 10.1186/1743-422X-9-16

**Published:** 2012-01-13

**Authors:** Jin-Yong Zhou, Le Zhang, Lei Li, Guang-Yu Gu, Yi-Hua Zhou, Jun-Hao Chen

**Affiliations:** 1 Department of Laboratory Medicine, Nanjing Drum Tower Hospital, Nanjing University Medical School, Nanjing, China; 2 Jiangsu Key Laboratory for Molecular Medicine, Nanjing University Medical School, Nanjing, China

## Abstract

**Background:**

Persistent hepatitis B virus (HBV) infection is a risk factor for hepatocellular carcinoma (HCC) development. This study aimed to clarify whether the high HBV DNA level is associated with HCC development by comparing HBV DNA levels between HBV infected patients with and without HCC.

**Results:**

There were 78 male and 12 female patients in each group and there was no statistical difference between these two group patients' average ages. The HBV DNA level in the HCC patients was 4.73 ± 1.71 Log_10 _IU/ml while 3.90 ± 2.01 Log_10 _IU/ml in non-HCC patients (*P *< 0.01). The HBeAg positive rate was 42.2% (38/90) in the HCC group while 13.3% (12/90) in the non-HCC group (*P *< 0.001). Compared with patients with HBV DNA level of < 3 Log_10 _IU/ml, the patients with level of 3 to < 4, 4 to < 5, 5 to < 6, or ≥ 6 Log_10 _IU/ml had the odds ratio for HCC of 1.380 (95% CI, 0.544-3.499), 3.671 (95% CI, 1.363-9.886), 5.303 (95% CI, 1.847-15.277) or 3.030 (95% CI, 1.143-8.036), respectively.

**Conclusions:**

HBV-related HCC patients had higher HBV DNA level than non-HCC counterparts. Our findings imply that active HBV replication is associated with the HCC development.

## Background

Chronic hepatitis B virus (HBV) infection is a serious public health problem worldwide since more than 350 million people are chronic carriers [1-4]. Persistent HBV infection is a risk factor for the development of hepatocellular carcinoma (HCC). The viral factors, such as viral genotype and mutants, have been shown to be associated with the pathogenesis of HCC [5-7]. Recent studies show that HBV load may also be associated with the HCC development [8-15] because the HBV DNA level in HCC patients was higher than that in non-HCC patients [13-15]. However, other studies showed that the viral load in HBV-associated HCC patients was not higher than that in the patients without HCC [16,17].

HBV infection is still highly endemic in China although the prevalence of hepatitis B surface antigen (HBsAg) has been reduced from 10% to 7.18% after the hepatitis B vaccine has been widely used in infants [18,19]. The age-standardized incidence of HCC in China is 58 per 100 000 persons for men and 22 per 100 000 persons for women, which is high in the world [20]. However, the comparison of HBV DNA level between HCC and non-HCC patients in China has been less studied [15]. In view of high HBV endemicity and HCC incidence in China, we performed this study to compare serum HBV DNA level between chronic hepatitis B (CHB) patients with HCC and the patients with HBV infection alone to clarify whether the high HBV level is a risk for HCC development.

## Results

### Patients' characteristics

In view of the fact that antiviral treatment will influence the HBV DNA level, we excluded the patients who received any antiviral treatment before. After these two group patients were matched for age (fewer than 2 years difference) and gender respectively, 90 patients were included in each group. Their average age, serum biochemical values and number of HBeAg positive patients are expressed in Table 1. Our findings showed the HBeAg positive rate was 42.2% (38/90) in the HCC group while 13.3% (12/90) in the non-HCC group (*P *< 0.001). As shown in Table 2, we found the percentages of patients with abnormal serum alanine aminotransferase (ALT), aspartate aminotransferase (AST), total bilirubin or direct bilirubin of HCC group were higher than those of non-HCC group. This indicates that higher percentage HCC patients are associated with worse liver function.

**Table 1:** Characteristics of HCC and non-HCC patients


**Characteristics**	**HCC (n = 90)**	**non-HCC (n = 90)**	**P value**

Male/Female	78/12	78/12	
Age	52.53 ± 10.27	52.53 ± 10.23	1.000
HBeAg positive	38	12	< 0.001
Total Protein (g/L)	68.2 ± 6.8	65.5 ± 8.1	0.017
Albumin (g/L)	38.5 ± 5.0	38.9 ± 6.4	0.657
Globulin (g/L)	29.7 ± 6.5	26.6 ± 5.6	0.001
ALT (IU/L)	47.50 (7.9-926.7)	25.05 (7.1-1660.5)	< 0.001
AST (IU/L)	48.15 (18.7-910.9)	26.35 (10.4-1071.1)	< 0.001
Total Bilirubin (μmol/L)	19.60 (3.6-503.9)	16.8 (6.7-492.7)	0.021
Direct Bilirubin (μmol/L)	7.30 (1.7-304.9)	5.15 (1.5-353.1)	< 0.001

**Table 2:** The percentage of patients with abnormal serum biochemical parameters


**Parameters**	**HCC (n = 90)**	**non-HCC (n = 90)**	**P value**

ALT	52 (57.8%)	21 (23.3%)	< 0.001
AST	53 (58.9%)	27 (30.0%)	< 0.001
Total Bilirubin	42 (46.2%)	29 (32.2%)	0.047
Direct Bilirubin	48 (53.3%)	24 (26.7%)	< 0.001
Total Protein	16 (17.8%)	21 (23.3%)	0.356
Albumin	23 (25.6%)	20 (22.2%)	0.600
Globulin	18 (20.0%)	14 (15.6%)	0.436

### Comparison of HCC and non-HCC patients' HBV DNA level

As shown in Table 3, we compared the viral load between HCC patients and non-HCC patients. The HBV DNA level was higher in HCC group than that in non-HCC group (4.73 ± 1.71 vs. 3.90 ± 2.01 log_10 _IU/ml, *P *< 0.01). We further analyzed the viral load of HCC or non-HCC patients stratified by age. The HBV DNA level in HCC group was significantly higher than that of the non-HCC patients in the age range of 50-59 years. This was the same with the age range of below 40 years.

**Table 3:** Comparison of the HCC and non-HCC patients' number and viral load stratified by ages


**Age range** ** (years)**	**Patients number (%)**			**viral load (Log_10 _IU/ml, mean ± SD)**		
	
	**HCC**	**non-HCC**	**P value**	**HCC**	**non-HCC**	**P value**

All	90 (100)	90 (100)	1.000	4.73 ± 1.71	3.90 ± 2.01	0.003
≥ 60	25 (27.8)	22 (24.4)	0.611	4.48 ± 1.80	3.56 ± 1.35	0.056
50-59	29 (32.2)	32 (35.6)	0.637	5.20 ± 1.52	3.98 ± 2.21	0.015
40-49	27 (30.0)	27 (30.0)	1.000	4.20 ± 1.85	4.20 ± 2.09	0.996
< 40	9 (10.0)	9 (10.0)	1.000	5.51 ± 0.94	3.61 ± 2.46	0.046

The odds ratios (ORs) for the association between HCC and various serum HBV DNA level are shown in Table 4. Compared with patients with HBV load of < 3 log_10 _IU/ml, patients with 3 to < 4, 4 to < 5, 5 to < 6, or ≥ 6 log_10 _IU/ml HBV DNA level had the ORs of 1.380 (95% CI, 0.544-3.499), 3.671 (95% CI, 1.363-9.886), 5.303 (95% CI, 1.847-15.277) or 3.030 (95% CI, 1.143-8.036), respectively. These results indicate that patients with serum HBV DNA level of above 10^4 ^IU/ml have a higher risk to develop HCC.

**Table 4:** Odds ratios for the association between HCC and various HBV load

HBV load (IU/ml)	HCC (n)	non-HCC (n)	OR (95% CI)	P value
≥ 10^6^	20	15	3.030 (1.143-8.036)	0.045

10^5 ^- < 10^6^	21	9	5.303 (1.847-15.277)	0.003

10^4 ^- < 10^5^	21	13	3.671 (1.363-9.886)	0.018

10^3 ^- < 10^4^	17	28	1.380 (0.544-3.499)	0.659

< 10^3^	11	25	1.000 (reference)	

## Discussion

We quantitatively measured serum HBV load in 90 HBV infected patients with HCC and in 90 age- and gender-matched patients with HBV infection alone and found that HCC patients had higher serum HBV DNA level than patients without HCC, especially in the age range of 50-59 years or below 40 years. The data in the present study indicate that the patients with higher HBV level have more risk to develop HCC.

There were a few retrospective studies in which the authors compared HCC and non-HCC patients' HBV DNA level to clarify the role of HBV load in HCC development. Some authors stated the level in HCC patients was higher than that in non-HCC patients [14,15] and > 10^4 ^[21] or > 10^5 ^[13] copies/ml HBV DNA level was supposed to be an independent risk factor of HCC development. As an indicator of active replication of HBV, the higher HBeAg positive rate is usually associated with an increased risk of HCC [22]. Our findings were similar to these previously reported results. However, in Tsai et al's paper, there existed no significant difference in HCC and non-HCC patients' HBV DNA level [16]. Another study performed in India showed that the HBV DNA load in HCC patients was lower than that of HBV-related chronic liver disease patients [17]. However, in these two studies, the HCC group patients were both obviously older than the non-HCC group patients (53.7 ± 12.7 vs. 32.1 ± 10.0 years [16], 53.6 ± 13.2 vs. 44.2 ± 14.7 years [17]). The evolution and the viral load of HBV infection may be influenced by age at acquisition. In the present study, although it is difficult to determine duration of the infection for each patient, we considered that most patients in our study had been infected with HBV in their infantile or childhood periods as the majority of chronic HBV infection in China is due to the perinatal infections [23]. Thus, the comparable patients' ages in the HCC and non-HCC groups may represent the similar infection durations in the two groups. Since HCC is a long-term outcome of chronic HBV infection and HBV DNA level is usually higher in younger CHB patients than in older ones [16], the matching of the patients' age is essential in the comparison of HBV DNA level in patients with or without HCC.

Large number chronic HBsAg carriers in Taiwan were followed up for a long period and the prospective data showed an increased risk for cirrhosis and for HCC with increasing level of HBV DNA [24,25]. Several studies have suggested an increased risk of HCC with high HBV DNA level (> 10^6 ^copies/ml [9], 10^4^-10^7 ^copies/ml [26] or > 10^5 ^copies/ml [27]). In our study, the data demonstrated that the patients with higher than 10^4 ^IU/ml HBV DNA level had more risk to be diagnosed as HCC, which was similar to the previous prospective reports [25-29]. The commonly used quantitative units of HBV DNA level in papers are IU/ml and copies/ml. In the current WHO HBV standard and consensus, one IU is approximately equivalent to five genome equivalents (copies) [30]. The viral load of 10^4 ^IU/ml identified in this study is approximately equal to 5 × 10^4 ^copies/ml.

## Conclusions

In summary, our findings demonstrated that higher serum HBV load in HCC patients than non-HCC counterparts, especially in the age range of both 50-60 years and below 40 years. Our results suggest that patients with higher than 10^4 ^IU/ml HBV DNA level are associated with more risk to develop HCC.

## Methods

### Patients and study design

A total of 388 CHB patients were admitted to Nanjing Drum Tower Hospital between October 2009 and June 2010. All patients were positive for HBsAg for at least 6 months. One group included 120 CHB patients who were diagnosed with HCC for the first time. The diagnosis of HCC was made by pathological findings or a combination of elevated α-fetoprotein (> 400 ng/ml) and typical appearance on at least two radiological imaging techniques, including ultrasonography, computerized tomography, magnetic resonance imaging, or hepatic angiography with lipiodol. The other group was 268 CHB patients without HCC. The patients with hepatitis C virus (HCV) or HIV co-infection, primary biliary cirrhosis, autoimmune hepatitis, significant alcohol intake or Wilson's disease were excluded. After we excluded the patients who received any antiviral treatment before, these two group patients were matched for age (fewer than 2 years difference) and gender respectively. As a result, there were 90 patients in each group. All study participants provided informed written consent. All the experiments were approved by the Ethics Committee of Nanjing Drum Tower Hospital, Nanjing University Medical School, in accordance with guidelines of the Nation Health and Medical Research Council of China.

A blood sample was collected from each subject and the serum was separated by centrifugation at room temperature. The serum was stored in a sterile tube at -20°C until use. For HCC patients, serum samples were collected before surgical resection or interventional therapy was performed.

### Viral markers assays

HBsAg and hepatitis B e antigen (HBeAg) were tested by commercial enzyme-linked immunosorbent assays (ELISA) (Huakang Biotechnology, Shenzhen, China). Commercial ELISA kit for anti-HCV was obtained from Huakang (Huakang Biotechnology, Shenzhen, China) and anti-HIV ELISA kit was from Lizhu (Lizhu Biotechnology, Zhuhai, China).

### Quantitative assay of HBV DNA

Serum HBV DNA was quantified with a commercial fluorescent real-time PCR assay (DaAn Gene, Guangzhou, China) [31]. Serum DNA was extracted from 100 μl serum by boiling in DNA extract buffer (NaOH, Tris-HCl, TritonX-100, NP40, chelex-100, EDTA) and was dissolved in 20 μl DNA solution buffer. Afterwards, 2 μl DNA solution was subjected to PCR. The PCR program consisted of 93°C for 2 min, 10 cycles of 93°C for 45 seconds and 55°C for 60 seconds, 30 cycles of 93°C for 30 seconds and 55°C for 45 seconds. The fluorescence signal of the amplicons was detected in every step of 55°C for 45 seconds by 7500 real time PCR system (Applied Biosystems, Singapore). The lower detection limit of this assay was 10^3 ^IU/ml with a linear range of up to 10^7 ^IU/ml. The known amounts of 10^3^, 10^4^, 10^5^, 10^6^, 10^7 ^IU/ml HBV DNA were used as a control. Strict precautions were taken to avoid potential contamination.

### Statistical analysis

Serum HBV DNA level was log_10_-transformed for analysis. Normal distribution data were expressed as mean ± standard deviation and non-normal distribution data were given as median with range. All the HBV DNA levels below the detection limit (3 log_10 _IU/ml) were defined as non-measured values and they were arbitrarily estimated as 1.5 log_10 _IU/ml in analysis for the reason of estimated continuous distribution of the HBV DNA levels. The differences in liver function and serum DNA level between HCC and non-HCC patients were evaluated by Pearson's Chi-square test, Student's *t *test and Mann-Whitney *u *test where appropriate. All estimates were accompanied by a 95% confidence interval (CI) where appropriate and two-sided *P *values of fewer than 0.05 were considered statistically significant.

## Competing interests

The authors declare that they have no competing interests.

## Authors' contributions

JYZ collected the samples, performed the experiments, analyzed the data, and drafted the manuscript. LZ and LL assisted in collecting the samples and measuring the HBV DNA. GYG interpreted the data and revised the manuscript. YHZ and JHC designed the study, interpreted the data and critically revised the manuscript. All authors read and approved the final manuscript.
